# Attempted DNA extraction from a Rancho La Brea Columbian mammoth (*Mammuthus columbi*): prospects for ancient DNA from asphalt deposits

**DOI:** 10.1002/ece3.928

**Published:** 2014-01-11

**Authors:** David A Gold, Jacqueline Robinson, Aisling B Farrell, John M Harris, Olaf Thalmann, David K Jacobs

**Affiliations:** 1 Department of Ecology and Evolutionary Biology, University of CaliforniaLos Angeles, California 90095; 2 The George C. Page Museum of La Brea Discoveries5801 Wilshire Boulevard, Los Angeles, California 90036; 3 Department of Biology, Division of Genetics and Physiology, University of TurkuItäinen Pitkäkatu 4, Turku 20014, Finland

**Keywords:** Ancient DNA, Columbian mammoth, La Brea tar pits, Rancho La Brea

## Abstract

Fossil-bearing asphalt deposits are an understudied and potentially significant source of ancient DNA. Previous attempts to extract DNA from skeletons preserved at the Rancho La Brea tar pits in Los Angeles, California, have proven unsuccessful, but it is unclear whether this is due to a lack of endogenous DNA, or if the problem is caused by asphalt-mediated inhibition. In an attempt to test these hypotheses, a recently recovered Columbian mammoth (*Mammuthus columbi*) skeleton with an unusual pattern of asphalt impregnation was studied. Ultimately, none of the bone samples tested successfully amplified *M. columbi* DNA. Our work suggests that reagents typically used to remove asphalt from ancient samples also inhibit DNA extraction. Ultimately, we conclude that the probability of recovering ancient DNA from fossils in asphalt deposits is strongly (perhaps fatally) hindered by the organic compounds that permeate the bones and that at the Rancho La Brea tar pits, environmental conditions might not have been ideal for the general preservation of genetic material.

## Introduction

Ancient DNA researchers are witnessing an unprecedented accumulation of genomic data, thanks to rapid technological advances such as multiplex PCR (Krause et al. 2005; Römpler et al. 2006) and next-generation sequencing (Margulies et al. 2005; Briggs et al. 2009; Avila-Arcos et al. 2011; Bos et al. 2011; Mason et al. 2011; Meyer et al. 2012; Paijmans et al. 2012). Unfortunately, even the most advanced methods require DNA preservation, and the ability to predict *a priori* which samples will provide endogenous DNA remains elusive. Researchers are debating the upper limits to the age at which ancient DNA remains viable, but there is agreement that certain conditions, such as temperature, can slow down or accelerate the degradation of genetic material (Hofreiter et al. 2001; Willerslev and Cooper 2005; Allentoft et al. 2012; Dabney et al. 2013; Orlando et al. 2013). Still, attempts to predict the quality of DNA in ancient samples using proxies such as relative age (Willerslev and Cooper 2005), environmental conditions (Letts and Shapiro 2012), or aspartic acid racemization (Poinar et al. 1996; Collins et al. 2009) have proven ambiguous. Ultimately, a better record of successes and failures in DNA extraction could help us discover general trends regarding genetic preservation in prehistoric samples.

Fossil-bearing asphalt deposits provide a unique mode of preserving ancient tissues, and in principle could provide a valuable source of ancient DNA. For example, the asphalt seeps of Rancho La Brea in Los Angeles, California, provide one of the world's richest deposits of Late Pleistocene biota, with over a million bones recovered representing at least 231 vertebrate species (Akersten et al. 1983; Shaw and Quinn, 1986; Stock and Harris 1992). For approximately the last 40,000 years, these seeps have episodically trapped organisms ranging from mammoths and saber-tooth cats to insects and juniper seeds. The majority of bones are not significantly weathered or modified by scavengers (reviewed in Harris 2001), although animal carcasses could remain exposed for months before being fully submerged in the asphalt (Holden et al. 2013). The asphalt itself provides an anoxic, hydrophobic environment, theoretically limiting aqueous-mediated diagenesis and preserving the bones to a level where they can be radiometrically dated and subjected to stable isotope analysis (Marcus and Berger, 1984; Coltrain et al. 2004; Ward et al., 2005; O'Keefe et al. 2009), suggesting conditions could be favorable for DNA preservation.

Several attempts have been made to extract DNA from Rancho La Brea fossils, but only one study has reported a positive result, DNA from the saber-tooth cat *Smilodon fatalis* (Janczewski et al. 1992). Phylogenetic analysis of a 132-base pair mitochondrial 12S sequence suggested that *S. fatalis* was a member of the modern cat clade. But a more recent study, which recovered significantly more sequence information from several Patagonian specimens of *Smilodon populator*, suggests that *Smilodon* is instead a sister taxon to living felids, which is consistent with traditional morphological studies that place *Smilodon* within the extinct felid subfamily *Machairodontinae* (Barnett et al. 2005). This suggests that the *S. fatalis* DNA from Janczewski et al. was the result of contamination (NCBI BLASTn (Altschul et al. 1990) finds a single-nucleotide mismatch between the Janczewski et al. *S. fatalis* sequence and the domestic cat *Felis catus*). The original study has not been replicated or followed up, and the general consensus has been that the Rancho La Brea asphalt, which permeates through most samples, is inhibitory to DNA extraction, assuming DNA is even preserved.

During the 2006 construction of an underground parking structure for the Los Angeles County Museum of Art, a nearly complete Columbian mammoth (*Mammuthus columbi*) skeleton was recovered, which showed unusual aspects to its preservation. Mammoths are relatively rare in the La Brea biota; remains of only 36 individuals have been identified in the collections from all 20th century excavations, and all individuals are only known from isolated elements. The new skeleton, named “Zed” by the Page Museum laboratory staff, is relatively complete, including the association of most postcranial skeletal elements and the preservation of both tusks. Collagen in examined bones is poorly preserved, but enough collagen was extracted to radiocarbon date Zed to 36,770 ± 750 BP (B. Fuller and J. Southon, pers. comm.). The skeleton was not recovered from a seep, although many of the bones were impregnated with asphalt. This offered a unique opportunity to tease apart the various hypotheses as to why ancient DNA extractions have previously failed in Rancho La Brea samples, and was the impetus for this project. Ultimately, we conclude that the environment in the Late Pleistocene southern California was probably not conducive for the preservation of ancient DNA, and even if it were, asphaltic contamination inhibits extraction from asphalt-permeated specimens using current extraction techniques.

## Methods and Materials

### Bone collection and preparation

Two samples were collected from the Rancho La Brea *M. columbi*, representing the range of preservation across the skeletal remains. For the first sample, a core approximately 2.5 cm in diameter and 5 cm deep was removed from the right scapula (LACMP23-1969). Due to the large size of the scapula, the core was sampled at the George C. Page Museum; all subsequent bone extractions and downstream processing of bone material were performed at UCLA's ancient DNA facility. At the time of sampling, the bone was still embedded in its protective plaster jacket; a small hole was cut through the jacket, and the exposed bone surface was wiped down with ethanol before sampling. While visual inspection revealed that the amount of asphalt in the scapula core was significantly less than what is typically found in Rancho La Brea specimens, trace amounts of asphalt were still present. Three different samples were prepared from the core material, to test the effects of varying degrees of asphalt contamination. In the first preparation, bone from the core was removed with a handheld Dremel tool and powdered with a mortar and pestle, without concern for asphalt contamination. In the second extraction, pieces of hardened asphalt were manually removed from the sample before being powdered. Finally, a third sample of powdered bone was cleaned using a protocol designed for radiocarbon-dating samples from Rancho La Brea (O'Keefe et al. 2009). 100–125 mg of powdered bone was washed in a series of petroleum ether, acetone, and finally hexane, for 5 min each. The sample was agitated during each wash, and the process was repeated a total of five times. During the fifth cycle, the powdered bone stayed in each reagent for 2 h, after which the sample was left to air dry in a fume hood. To test the effect of this cleaning process on the integrity of DNA, bone powder generated from a modern chicken femur was also prepared using this cleaning technique.

The second bone sample was removed from a rib fragment (LACMP23-554). While this rib had no visible exposure to asphalt, it did show significant permineralization and had spent considerable time in the laboratory of the George C. Page Museum, where it was potentially exposed to human and animal DNA (including the remains of a modern elephant housed in the museum's comparative collection). To minimize potential contamination, the surface of the bone was wiped down with bleach and then placed under a UV light for 15 min. The surface of the bone was removed using a Dremel tool, and a portion of the interior was processed for DNA extraction.

### DNA extraction

All bone samples were extracted using an ancient DNA-specific protocol, based on Rohland et al. (2010a) with some modifications. For these extractions, 100–250 mg of powdered sample was combined with 5 mL of extraction buffer (for 50 mL solution: 45 mL 0.5M EDTA pH 8.0, 1.25 mL proteinase K (10 mg/mL), and 3.75 mL ddH_2_O). The sample was shaken and left overnight on a rotator. The next day, the samples were centrifuged for 2 min at 4000 rpm. The supernatant was placed in a fresh falcon tube, and 0.5 volumes of binding buffer was added (for 50 mL solution: 29.5 g guanidine thiocyanate, 5 mL 3 mol/L sodium acetate, and 23 mL ddH_2_O). 100 *μ*L of the silica suspension buffer was then added. The supernatant, binding buffer, and silica suspension were left on a rotator for 3 h in the dark and were then centrifuged for 2 min at 4000 rpm. The supernatant was discarded, and the silica pellet was resuspended in 1 mL of binding buffer. The suspension was transferred to a new tube and centrifuged at maximum speed for 15 sec. The supernatant was discarded, and the pellet was resuspended in 1 mL wash buffer (for 50 mL solution: 25.65 mL ethanol, 1.25 mL sodium chloride, 0.5 mL 1 mol/L Tris pH 8.0, 100 *μ*L 0.5 mol/L EDTA pH 8.0, and ddH_2_O to 50 mL). The sample was then centrifuged at maximum speed for 20 sec and resuspended in the wash buffer a second time, followed by centrifugation at maximum speed for 20 sec, after which all supernatant was removed. The pellet was dried for 5 min under a fume hood, and 50 *μ*L of TE buffer was added. The sample rested for 10 min in buffer, before being centrifuged at maximum speed for 2 min. 48 *μ*L was placed in a new tube, being careful to avoid silica, and this sample was used for downstream amplification processes. For each bone sample preparation, at least two independent extractions were attempted.

In addition to the ancient DNA extraction protocol, we performed three phenol–chloroform extractions (modified from Sambrook and Russell 2001) on each of the two mammoth bone samples, and on a modern chicken femur for a positive control. For each extraction, a small piece of bone (approximately 5 mm × 5 mm) was homogenized in 1 mL of extraction buffer (100 m mol/L Tris/HCL (pH 5.5), 10 m mol/L EDTA, 0.1 mol/L NaCl, 1% SDS, and 1% *β*-mercaptoethanol). The samples were digested with proteinase K for 10 min at 55°C and then placed on ice. 11 *μ*L of 0.2 mol/L sodium acetate (pH ≈ 4) and 250 *μ*L of a freshly prepared phenol:chloroform:isoamyl (25:24:1) solution were added to each sample and left on ice for 15 min. Samples were centrifuged for 15 min at 10,000 rpm and 4°C. The upper phase was transferred into a new tube, 1 volume of isopropanol was added, and the samples were placed in a −80°C freezer overnight. This upper phase was centrifuged for 15 min (14,000 rpm, 4°C), the supernatant was removed, and the pellet was washed in 70% EtOH. The pellet was centrifuged once more for 5 min (9000 *g*, 4°C), before being dried and resuspended in ddH2O.

### DNA amplification

The first amplification method, which was applied to the scapula sample, involved a multiplex technique (Römpler et al. 2006). This methodology is well suited for simultaneously amplifying a large number of loci from limited and highly degraded DNA samples (Arandjelovic et al. 2009). It involves designing a series of primer pairs approximately 100 base pairs long, which produce overlapping PCR products. In the first amplification, nonoverlapping PCR products are pooled together in a multiplex reaction. A second round of PCR – the simplex reaction – is then performed on the individual primer pairs (see also Thalmann et al. 2011).

PCR primers were designed using the mitochondrial gene *cytochrome b* from the American mastodon (*Mammut americanum*), wooly mammoth (*Mammuthus primigenius*), Asian elephant (*Elephas maximus*), and African elephant (*Loxodonta africana*). These sequences were aligned with human *cytochrome b*, and a region was chosen that showed high conservation between proboscideans to the exclusion of humans. Five primer pairs were ultimately designed to target a 431-base pair region (see Data S1). Although the *M. columbi* mitochondrial genome reported in Enk et al. (2011) was not publically available at the time these primers were designed, *M. columbi cytochrome b* sequences show no significant divergence from the other proboscideans; the priming sites are free of polymorphisms, and positive amplification results would be expected using the designed primers.

Two microliters of each PCR primer (100 *μ*mol/L each) was combined into one of two multiplex primer mixes; the first primer mix included primer pairs #1, #3, and #5, whereas the second mix contained pairs #2 and #4. Water was added to each multiplex primer mix to bring the final volume to 200 *μ*L. A master mix was then created by combining 1x GeneAmp PCR Buffer (Invitrogen, Grand Island, NY; SKU# N8080010), 1 mg/mL bovine serum albumin (BSA), 4 m mol/L MgCl_2_, 0.25 m mol/L dNTPs (each), 2U of AmpliTaq Gold (Invitrogen, SKU# 4318739), 5 *μ*L of DNA, and 2.4 *μ*L of water per sample. Finally, 3 *μ*L of each multiplex primer mix was combined with 12 *μ*L of the multiplex master mix. The multiplex reactions were amplified using the following PCR protocol: 94°C for 9 min, followed by 30 cycles of 94°C for 20 sec, 57°C for 30 sec, and 72°C for 30 sec, followed by a final elongation of 72°C for 4 min.

In the simplex reactions, individual primer pairs were tested against the multiplexed products. For each primer pair, 20 *μ*L of the forward primer and reverse primer was mixed, and 160 *μ*L of water was added. The PCR master mix was identical to the multiplex reaction, with the exception that 1 *μ*L of BSA and 0.05 *μ*L of AmpliTaq Gold were used. PCR proceeded as described in the multiplex reaction, meaning that each product was amplified for 60 cycles total.

In addition to the designed multiplex primers, the scapula samples were tested using highly conserved 12S primers (12Sa and 12So, described in Poinar et al. 1998) that amplify a 153-base pair product (we designate these as “universal 12S” primers for the rest of this article). PCR was performed as described above, but with 50 cycles. For the mammoth samples, a series of dilutions were also performed (0.5×, 0.2×, 0.1×, 0.02×, and 0.001×).

Because we were not limited in material for the rib extraction, we abandoned the multiplex technique for a traditional PCR approach. We designed two sets of primers to amplify a 70-base pair region and a 120-base pair region of *cytochrome b*, using the same alignment as before (see Data S1). We also tested the 12S primers as described above. PCR products were run in triplicates and cloned using the TOPO TA Cloning Kit with the PCR 2.1-TOPO Vector, following the manufacturer's protocol. Sequencing was performed at Cornell University's Life Sciences Core Laboratories Center, using the Applied Biosystems (Foster City, CA) Automated 3730 DNA Analyzer.

### DNA spiking experiments

To test for the presence of inhibitory compounds in the La Brea asphalt, we performed a series of “spiking” experiments, mixing DNA from the positive control (chicken femur) with the mammoth samples. In one set of experiments, we combined mammoth and chicken bone powder together before using one of the two extraction protocols (ancient DNA or phenol–chloroform) described previously. In the second series of experiments, we spiked the mammoth samples with chicken DNA following extraction but before PCR amplification, using dilutions of 25%, 50%, and 75% mammoth DNA to chicken DNA.

## Results

**Figure 1:**
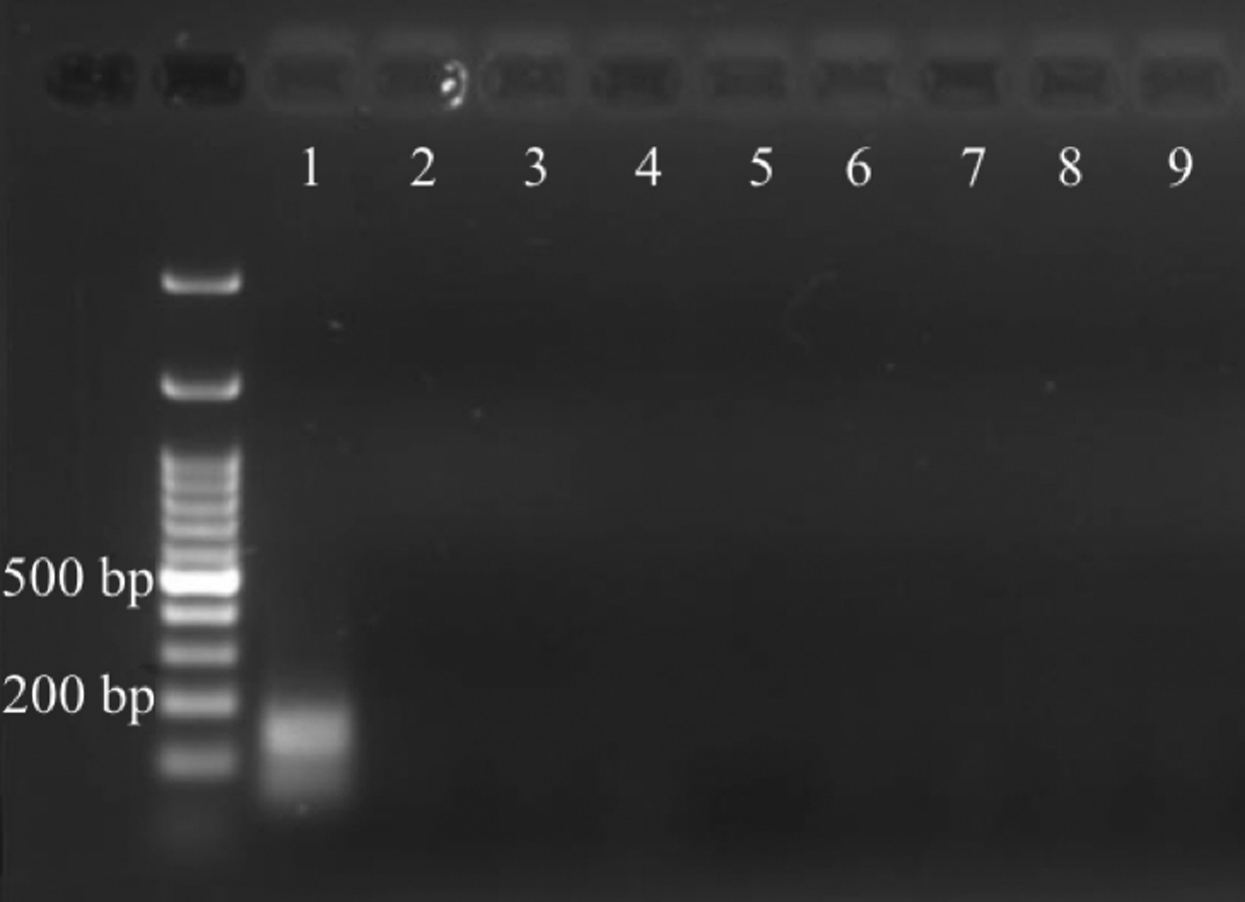
Gel image summarizing the results from PCR amplification of mitochondrial 12S DNA from chicken and mammoth samples. (1–3) Phenol–chloroform protocol: (1) chicken femur, (2) *Mammuthus columbi* scapula, (3) *M. columbi* rib. (4–8) Ancient DNA protocol: (4) *M. columbi* scapula, (5) scapula with visible asphalt removed, (6) scapula cleaned with hexane, petroleum ether, and acetone, (7) *M. columbi* rib (8) chicken femur cleaned with hexane, petroleum ether, and acetone. (9) Negative control including all primers used in this study.

The samples used, methods employed, and final results are summarized in Table 1. None of the samples extracted from the scapula core amplified PCR products, including both the primers designed to target mammoth DNA, as well as the universal 12S primers. The rib fragment did amplify the mammoth-specific “70-bp” and “120-bp” PCR products, but they were not the predicted size. When these PCR products were sequenced, they did not match animal mitochondrial DNA, but instead showed low e-value support against a small number of microbes (using NCBI's BLASTn program). We could not amplify the mitochondrial 12S sequence from the rib fragment either, suggesting that if any endogenous DNA fragments remain they are either less than 153 bp long or are in some way damaged and resistant to amplification. A summary of the 12S PCR amplifications is visualized in Fig. 1.

**Table 1:** Overview of the experiments attempted in this study and the results.

Sample	Preparation of material	Method of extraction	Color of extraction buffer after sample added	PCR method(s)	Primers	PCR product(s)
Scapula	Raw sample powdered	Ancient DNA protocol	Black	Multiplex PCR; Standard PCR	Multiplex mammoth primers (Data S1); 12S primers	No; No
	Visible asphalt removed before powdering	Ancient DNA protocol	Yellow	Multiplex PCR; Standard PCR	Multiplex mammoth primers; 12S primers	No; No
	Visible asphalt removed before powdering	Phenol–chloroform protocol	N/A	Standard PCR	12S primers	No
	Sample cleaned after powdering	Ancient DNA protocol	Clear	Multiplex PCR; Standard PCR	Multiplex mammoth primers; 12S primers	No; No
	Visible asphalt removed, spiked with powdered chicken bone before extraction	Ancient DNA protocol; phenol–chloroform protocol	Yellow; N/A	Standard PCR	12S primers	No; No
	Visible asphalt removed, spiked with chicken DNA after extraction and before PCR	Ancient DNA protocol; phenol–chloroform protocol	Yellow; N/A	Standard PCR	12S primers	Yes; Yes
Rib	Raw sample powdered	Ancient DNA protocol	Clear	Standard PCR	“120-bp” and “70-bp” primers; 12S primers	Yes (but wrong products); No
	Raw sample powdered	Phenol–chloroform protocol	N/A	Standard PCR	12S primers	No

The most informative results came from the “spiking” experiments, and our attempts to amplify DNA from a modern chicken femur. Although 12S DNA was readily amplified from chicken bone using a standard phenol–chloroform extraction, when the same material was cleaned using the protocol designed to remove asphalt (see methods section for details), PCR amplification failed (lane 8 in Fig. 1). This suggests that the reagents typically used to clean Rancho La Brea specimens either remove DNA from samples or otherwise inhibit DNA extraction/amplification using either extraction method. In our “spiking” experiments, we tested our ability to amplify chicken DNA when it was combined with mammoth scapula DNA, either before or after DNA extraction. When chicken DNA was independently extracted and added to mammoth DNA prior to PCR amplification, we were able to recover our universal PCR products across all dilutions. However, if the chicken bone was combined with the mammoth material before DNA extraction, none of the extractions amplified a PCR product. This strongly suggests that chemicals present in the mammoth bone material inhibit the extraction of DNA, but that these chemicals are either not carried over into the final DNA extracts or do not act as inhibitors of PCR.

## Discussion

Ancient DNA is notoriously difficult to work with; extracting genetic material from a fossil is unpredictable and never guaranteed. The results from this study suggest that a major hurdle to any future attempt at extracting DNA specimens recovered from Rancho La Brea, or any fossil-bearing asphalt deposit, will be finding a way to remove the asphalt without removing or damaging DNA. Even the minimally asphaltic samples taken from the scapula discolored the extraction buffer (see Table 1), which could be indicative of enzymatic inhibitors of downstream processes. Cleaning the bone powder also appears to inhibit DNA extraction, as evidenced from the modern chicken bone. Our results suggest that washing the samples through a series of petroleum ether, hexane, and acetone precluded DNA amplification, although the reasons are not entirely clear. The reagents used in this study were all organic solvents (petroleum ether and hexane are nonpolar organic hydrocarbons, whereas acetone is a polar organic ketone), and it is conceivable that one or more of them leached DNA from the samples. However, organic solvents – particularly phenol and chloroform – are commonly used in phase separation to denature proteins and preferentially retain DNA at low pH values (Sambrook and Russell 2001). Additionally, all three solvents have been successfully applied to cleaning asphalt-contaminated, or otherwise polluted, activated sludge samples for metagenomic analyses (e.g., Purohit et al. 2003). One possible explanation of this discrepancy is that in activated sludge samples, cleaning occurs before cellular lysis, so DNA should not be directly exposed to the organic solvents. However, in ancient tissue samples, cells are no longer intact, so it is possible that ancient DNA, if it existed, was in direct contact with these reagents. Although we had no *a priori* expectation that the reagents used would inhibit DNA extraction, the results from this study suggest that this cleaning technique should be avoided in preparing asphalt-permeated bone for future ancient DNA analyses.

Additionally, negative results from the rib fragment suggest that the environment of southern California in the Late Pleistocene was not ideal for the preservation of genetic material. Information from pollen analyses and deep-sea cores off the coast of southern California suggest the region was generally cooler than present during the last 50,000 years (Heusser 1998) and was dominated by woodland and chaparral. Although highly fragmented DNA can survive in such climates for hundreds of thousands of years (Dabney et al. 2013), ancient DNA recovered from mammoths and mastodons has generally come from specimens preserved in permafrost (Rogaev et al. 2006; Barnes et al. 2007; Haile et al. 2009; Rohland et al. 2010b; Cappellini et al. 2012; Nyström et al. 2012). Ancient DNA has been reported from proboscideans not preserved in permafrost, but several of these studies have been seriously challenged (e.g., see the critique by Rohland et al. (2007) of DNA recovered from a Michigan mastodon fossil (Yang et al. 1996), or challenges by Binladen et al. (2007) and Orlando et al. (2007) to claims of DNA recovered from a Cretan pygmy elephant fossil (Poulakakis et al. 2006)). The geographic range of *M. columbi* was significant, extending across the Southwestern United States into Florida and Mexico, but it appears to have stayed south of the North American ice sheets (Graham 2001). Subsequently, no permafrost specimens of *M. columbi* have been recovered. However, Enk et al. (2011) reported mitochondrial DNA from two *M. columbi* individuals. The first was from a specimen discovered in Huntington Canyon, Utah, which had previously been shown to exhibit particularly exquisite collagen matrix preservation in bone and dentine tissue (Schaedler et al. 1992). The second specimen was recovered near Rawlins, Wyoming; DNA fragments recovered from the teeth of this sample were identical to the Huntington mammoth. Whether the dentine of the Rancho La Brea mammoth would prove a better prospect than postcranial bone is uncertain. The skull of the Rancho La Brea mammoth (which was permeated with asphalt) was not available at the time the scapula or rib samples were taken, but it has since been prepared in the laboratory at the George C. Page Museum. Given the degree of diagenesis in the rib, an asphalt-free bone with less permineralization would be useful in further testing our hypothesis that the environment was not conducive to ancient DNA preservation.

Finally, the recovery of what appears to be noneukaryotic DNA from the rib fragment highlights the issue of microbial contamination. It is possible that contamination occurred during the bone's handling in the museum collections, but it is also known that Rancho La Brea asphalt supports dense communities of petroleum-reducing bacteria and archaea, many of which were previously unknown to science before their discovery in the tar pits (Zhao et al. 1989; Kim and Crowley 2007). The genome of *Methanocorpusculum labreanum*, a methanogenic archaeon that has only been described from Rancho La Brea (Zhao et al. 1989), was sequenced by the Joint Genome Institute (Anderson et al. 2009). We compared our recovered DNA sequences to the *M. labreanum* genome using BLASTn, but did not find any significant similarity.

Ultimately, prospects for retrieving ancient DNA from Rancho La Brea samples appear hindered by (1) the inability to remove asphalt from samples without also inhibiting DNA extraction, (2) diagenesis in nonasphaltic samples, and (3) the abundance of associated microbes. Because removing hydrophobic hydrocarbons, such as asphalt, requires organic solvents, it is not clear what sort of cleaning process would be preferable to the one currently employed by researchers working on Rancho La Brea specimens. However, a continued search for samples that have limited asphalt impregnation, particularly tooth and tusk specimens, might prove more promising. Additionally, while our extractions did not provide enough quality starting material for high-throughput shotgun sequencing, some combination of target enrichment with a next-generation sequencing approach might help resolve whether low amounts of highly fragmented mammoth DNA exist.

## Supporting Information

**Data S1.** Nucleotide alignment of partial proboscidean and human *cytochrome b* sequences, highlighting the primers designed.
