# Supplementary material for: Attempted DNA extraction from a Rancho La Brea Columbian mammoth (Mammuthus columbi): prospects for ancient DNA from asphalt deposits
Source: Ecol Evol. 2014 Jan 11;4(4):329–36. doi: 10.1002/ece3.928 (PMC3936381; doi:10.1002/ece3.928)
Supplement: Supplementary file 1 — Data S1. Nucleotide alignment of partial proboscidean and human cytochrome b sequences, highlighting the primers designed. [file ece30004-0329-sd1.docx]

**Supplement 1**: Nucleotide alignment of partial proboscidean and human *cytochrome b* sequences, highlighting the primers designed. The taxa numbers in the alignment represent (1) *Mammut americanum* (gi|148540928), (2) *Loxodonta africana* (gi|218783279), (3) *Elephas maximus* (gi|156938349), (4) *Mammuthus primigenius* (gi|157367496), (5) *Mammuthus columbi* (gi|333236151), and (6) *Homo sapiens* (gi|229503546). Forward multiplex primers are highlighted in yellow, reverse multiplex primers are highlighted in pink. *M. columbi* specific forward primers designed for the rib fragment are highlighted in blue, reverse primers in green.

1 TTAATGACCCACATCCGAAAATCCCATCCTCTATTTAAAATCATTAACAAGTCTTTCATT

2 ATAATGACCCACATTCGAAAATCTCACCCCTTACTTAAAATCATCAATAAATCCTTCATT

3 ATAATGACCCACACCCGAAAATCTCACCCCCTGTTTAAAATCATCAACAAATCCTTCATT

4 ATAATGACCCACATTCGAAAATCTCACCCCCTACTTAAAATCCTTAATAAATCCTTCATT

5 ATAATGACCCACATTCGAAAATCTCACCCCCTACTTAAAATCCTCAATAAATCCTTCATT

6 --AATGACCCCAATACGCAAAATTAACCCCCTAATAAAACTAATTAACCACTCATTCATC

1 GACTTACCCACCCCACCTAACATCTCAGCATGATGAAATTTCGGCTCACTACTAGGAGCA

2 GATCTACCTACCCCATCCAACATCTCAACATGATGAAATTTCGGCTCACTACTAGGAGCA

3 GATCTACCCACCCCATCTAACATCTCAACATGATGAAATTTCGGCTCACTACTAGGAGCG

4 GATCTACCTACCCCATCTAACATCTCAACATGATGAAATTTCGGCTCACTACTAGGAGCA

5 GATCTACCTACCCCATCTAACATCTCAACATGATGAAATTTCGGCTCACTACTAGGAGCA

6 GACCTCCCCACCCCATCCAACATCTCCGCATGATGAAACTTCGGCTCACTCCTTGGCGCC

1 TGTCTGATTACCCAGATCCTAACGGGGCTATTCCTAGCCATACACTACACACCCGATACA

2 TGCCTAATTACCCAGATCCTAACAGGATTATTCCTAGCCATACATTATACACCTGACACA

3 TGCCTAATTACCCAAATCCTAACAGGATTATTCCTAGCCATACATTACACACCTGACACA

4 TGCCTAATTACCCAAATCCTAACAGGGTTATTTCTAGCCATACATTATACACCTGACACA

5 TGCCTAATTACCCAGATCCTAACAGGGTTATTTCTAGCCATACATTATACACCTGACACA

6 TGCCTGATCCTCCAAATCACCACAGGACTATTCCTAGCCATGCACTACTCACCAGACGCC

1 ATAACCGCATTTTCATCTATATCCCACATCTGCCGAGACGTCAACTATGGCTGAACCATT

2 ATAACTGCATTTTCATCTATATCCCATATTTGCCGAGATGTGAACTACGGCTGAATTATT

3 ATAACTGCATTTTCATCTATATCCCATATCTGCCGAGACGTCAACTACGGCTGAATTATT

4 ATAACTGCATTTTCATCTATATCCCATATCTGCCGAGATGTCAACTACGGTTGAATTATT

5 ATAACTGCATTTTCATCTATATCCCATATCTGCCGAGATGTCAACTACGGTTGAATTATT

6 TCAACCGCCTTTTCATCAATCGCCCACATCACTCGAGACGTAAATTATGGCTGAATCATC

1 CGACAACTACACTCAAACGGAGCATCTATCTTCTTCCTATGTCTATACGCACACATTGGA

2 CGACAACTACACTCAAACGGAGCATCCATTTTCTTTCTCTGCCTATACACACACATTGGA

3 CGACAACTGCACTCAAACGGAGCATCTATCTTTTTCCTCTGCCTATACACACACATTGGA

4 CGACAACTACACTCAAACGGAGCATCTATTTTCTTCCTCTGCCTATACACACACATTGGA

5 CGACAACTACACTCAAACGGAGCATCTATTTTCTTCCTCTGCCTATACACACACATTGGA

6 CGCTACCTTCACGCCAATGGCGCCTCAATATTCTTTATCTGCCTCTTCCTACACATCGGA

1 CGAAACATCTACTATGGGTCCTACCTATACTCAGAAACCTGAAATACCGGCATTATACTA

2 CGAAACATCTACTATGGGTCCTACCTATACTCGGAAACTTGAAATACCGGCATTATATTA

3 CGAAACATCTACTATGGATCCTATCTATACTCAGAAACCTGAAACACAGGTATTATACTA

4 CGAAACATCTACTATGGGTCCTACCTATACTCGGAAACCTGAAATACCGGCATTATACTA

5 CGAAACATCTACTATGGGTCCTACCTATACTCGGAAACCTGAAATACCGGCATTATACTA

6 CGAGGCCTATATTACGGATCATTTCTCTACTCAGAAACCTGAAACATCGGCATTATCCTC

1 CTGATAATCACGATAGCTACTGCTTTCATGGGATATGTCCTCCCATGAGGACAGATATCA

2 CTACTAATCACCATAGCCACCGCCTTCATAGGATATGTCCTTCCGTGAGGACAAATATCA

3 CTACTAATCACCATAGCCACCGCCTTCATAGGATATGTCCTTCCATGAGGACAAATATCA

4 CTACTAATCACCATAGCCACCGCCTTCATAGGATATGTCCTTCCGTGAGGACAAATATCA

5 CTACTAATCACCATAGCCACCGCCTTCATAGGATATGTCCTTCCGTGAGGACAAATATCA

6 CTGCTTGCAACTATAGCAACAGCCTTCATAGGCTATGTCCTCCCGTGAGGCCAAATATCA

1 TTTTGAGGAGC

2 TTCTGAGGGGC

3 TTCTGAGGGGC

4 TTCTGAGGGGC

5 TTCTGAGGGGC

6 TTCTGAGGGGC
